# The Arg82Cys Polymorphism of the Protein Nepmucin Implies a Role in HDL Metabolism

**DOI:** 10.1210/jendso/bvac034

**Published:** 2022-03-04

**Authors:** Sophia Metz, Nikolaj T Krarup, Thomas Bryrup, Julie Støy, Ehm A Andersson, Christina Christoffersen, Matt J Neville, Malene R Christiansen, Anna E Jonsson, Daniel R Witte, Ulla Kampmann, Lars B Nielsen, Niklas R Jørgensen, Fredrik Karpe, Niels Grarup, Oluf Pedersen, Tuomas O Kilpeläinen, Torben Hansen

**Affiliations:** 1 Novo Nordisk Foundation Center for Basic Metabolic Research, Faculty of Health and Medical Sciences, University of Copenhagen, 2200 Copenhagen, Denmark; 2 Department of Cardiology, Aalborg University Hospital, 9000 Aalborg, Denmark; 3 Aarhus University Hospital, Steno Diabetes Center Aarhus, 8200 Aarhus, Denmark; 4 Department of Clinical Biochemistry, Rigshospitalet, 2100 Copenhagen, Denmark; 5 Department of Biomedical Sciences, Faculty of Health and Medical Sciences, University of Copenhagen, 2200 Copenhagen, Denmark; 6 Oxford Centre for Diabetes, Endocrinology & Metabolism, OX3 7LE Oxford, UK; 7 Oxford NIHR Biomedical Research Centre, Churchill Hospital, OX3 7LE Oxford, UK; 8 Department of Public Health, Section of Epidemiology, Aarhus University, 8000 Aarhus, Denmark; 9 Faculty of Health, Aarhus University, 8000 Aarhus, Denmark; 10 Institute of Clinical Medicine, Faculty of Health and Medical Sciences, University of Copenhagen, 2200 Copenhagen, Denmark; 11 Faculty of Health, University of Southern Denmark, 5000 Odense, Denmark

**Keywords:** lipid metabolism, cholesterol transport, human genetics, apolipoproteins, triglycerides, metabolism

## Abstract

**Context:**

Blood lipid levels are linked to the risk of cardiovascular disease and regulated by genetic factors. A low-frequency polymorphism Arg82Cys (rs72836561) in the membrane protein nepmucin, encoded by *CD300LG*, is associated with lower fasting concentration of high-density lipoprotein cholesterol (HDLc) and higher fasting triglycerides. However, whether the variant is linked to postprandial lipids and glycemic status remains elusive.

**Objective:**

Here, we augment the genetic effect of Arg82Cys on fasting plasma concentrations of HDL subclasses, postprandial lipemia after a standardized high-fat meal, and glycemic status to further untangle its role in HDL metabolism.

**Methods:**

We elucidated fasting associations with HDL subclasses in a population-based cohort study (Oxford BioBank, OBB), including 4522 healthy men and women. We investigated fasting and postprandial consequences on HDL metabolism in recall-by-genotype (RbG) studies (fasting: 20 carrier/20 noncarrier; postprandial: 7 carrier/17 noncarrier), and shed light on the synergistic interaction with glycemic status.

**Results:**

A lower fasting plasma concentration of cholesterol in large HDL particles was found in healthy male carriers of the Cys82 polymorphism compared to noncarriers, both in the OBB (*P* = .004) and RbG studies (*P* = .005). In addition, the Cys82 polymorphism was associated with low fasting plasma concentrations of ApoA1 (*P* = .008) in the OBB cohort. On the contrary, we did not find differences in postprandial lipemia or 2-hour plasma glucose levels.

**Conclusion:**

Taken together, our results indicate an association between the Arg82Cys variant and a lower concentration of HDL particles and HDLc, especially in larger HDL subclasses, suggesting a link between nepmucin and HDLc metabolism or maturation.

Dyslipidemia is a major risk factor for cardiovascular disease [1-5]. Higher blood levels of low-density lipoprotein cholesterol (LDLc) and triglycerides (TGs) are associated with an increased cardiovascular disease risk, whereas higher levels of high-density lipoprotein cholesterol (HDLc) are associated with a lower risk. The protective effect of HDLc has been primarily attributed to the beneficial role of HDLc in reverse cholesterol transport [6], whereby free cholesterol is transported via HDL particles from peripheral tissues to the liver, and cholesterol esters and TGs are exchanged between HDL and very low-density lipoprotein (VLDL), LDL, and other apolipoprotein B–containing particles [7, 8]. HDL incorporates a range of particle sizes with different properties for transporting circulating cholesterol [9]. The main HDL categories based on diameter include very large (XL) and large (L) particles, also called HDL2 particles, and medium (M) and small (S) particles, also called HDL3 particles. Particle size may affect the cardioprotective potential of HDL, with several studies suggesting that the cardioprotective characteristics are harbored by the smaller HDL3 particles [10-12]. However, the literature remains controversial in this regard [13].

Genome-wide association studies (GWAS) harbor great potential to identify new targets involved in HDL metabolism. In recent GWAS, the minor Cys82 polymorphism (frequency 3% in European ancestry) of the missense variant Arg82Cys (rs72836561) in *CD300LG* has been associated with lower fasting plasma HDLc [14-18] and higher plasma TG levels [14]. The Arg82Cys variant is predicted as “damaging” by structural prediction tools (Polyphen-2 and MuPred-2), indicating that the variant hampers CD300LG function [19, 20]. *CD300LG* encodes nepmucin [21], a protein enriched in capillary endothelial cells [22, 23]. The Arg82Cys polymorphism is located in the immunoglobulin (Ig) domain of nepmucin that reaches into the extracellular matrix. Nepmucin has been implicated in lymphocyte trafficking, in particular lymphocyte rolling and binding through its mucin-like and Ig-domain, as well as in transendothelial migration [24-26]. However, a role of nepmucin in lipid homeostasis remains unclear.

To shed light on the role of nepmucin in lipid homeostasis, we carried out population-based genetic association studies to investigate the nepmucin Arg82Cys polymorphism and fasting plasma HDL subclasses [27]. Furthermore, we performed recall-by-genotype (RbG) studies to detect additional subtle abnormalities in lipid metabolism that could not be detected in a fasting state alone, and studied synergistic interactions of Cys82 with glycemic status (study overview in Fig. 1).

**Figure 1.**
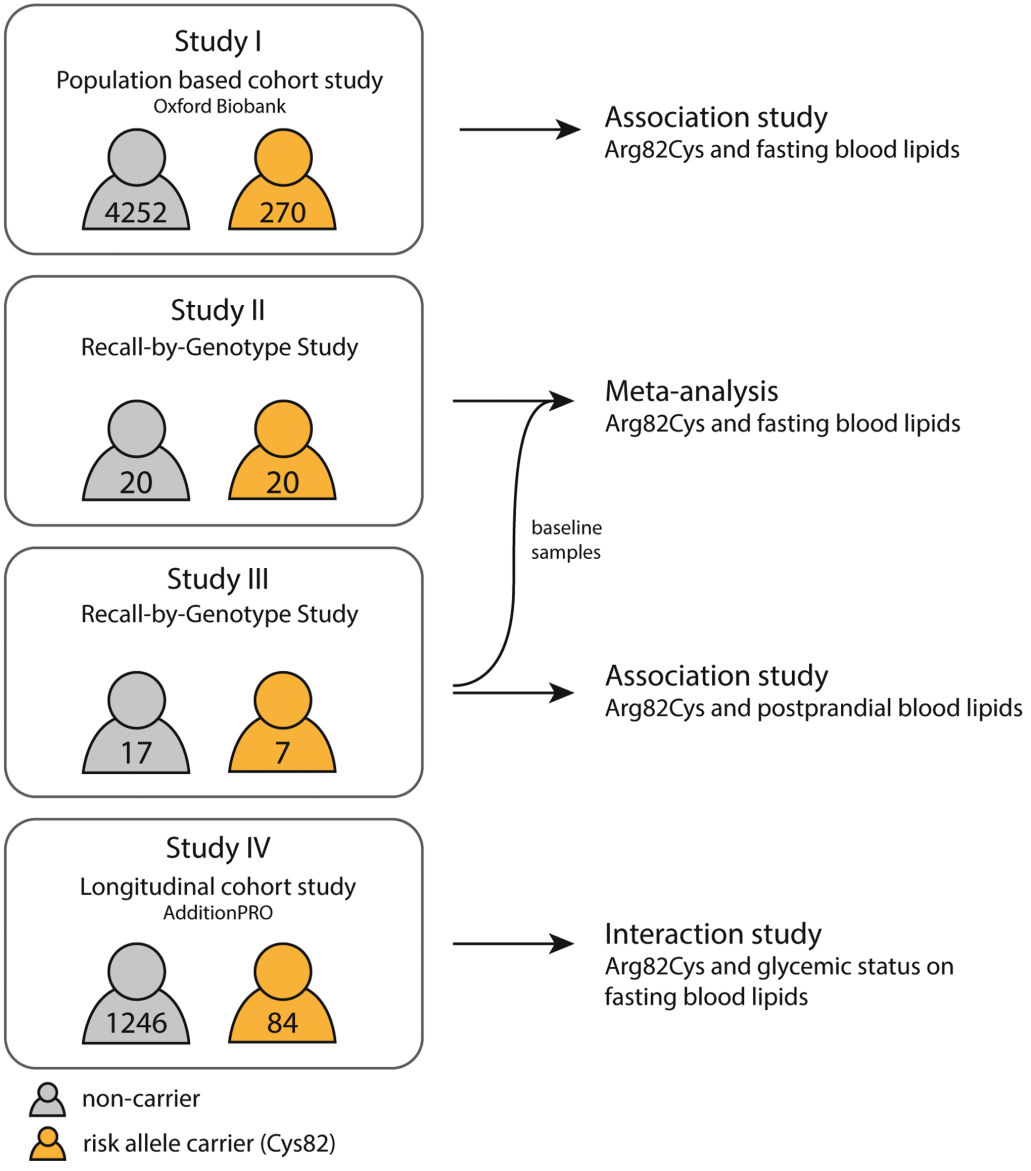
Study overview of the included studies I to IV. Schematic representation of the included studies and respective analysis. The included number of noncarriers is marked in gray, and heterozygous and homozygous risk-allele carriers are combined and presented in orange.

## Material and Methods

### Study I—Nepmucin Arg82Cys Variant and Fasting Plasma High-density Lipoprotein Subclasses in 4522 British Men and Women

HDL subclasses were measured by nuclear magnetic resonance (NMR)-spectroscopy in 4522 individuals from the Oxford BioBank (OBB) [28]. The OBB is a cohort of healthy, normoglycemic European ancestry men and women aged 30 to 50 years, randomly selected from the Oxfordshire area [29]. A high-throughput proton NMR platform containing approximately 230 metabolites has been performed on approximately 7100 OBB plasma samples [27, 28]. The quantified HDL subclasses are defined as XL-HDL (average particle diameter 14.3 nm), L-HDL (12.1 nm), M-HDL (10.9 nm), and S-HDL (8.7 nm). The NMR platform has been used in multiple epidemiological and genetic studies [30, 31], and the details of the method have been described elsewhere [27, 32].

### Study II—Nepmucin Arg82Cys Variant and Fasting Plasma Lipid Variables in 20 Noncarriers + 20 Carriers Combined in a Meta-analysis With 17 Noncarriers and 7 Carriers (From Study III)

Here, we performed a meta-analysis of the associations with fasting plasma HDL subclasses in 20 healthy male heterozygous carriers of the Cys82 variant and 20 matched noncarriers [33, 34] and the baseline/fasting results from study III (7 heterozygous carriers of Cys82, and 17 noncarriers). The 20 Cys82 carriers and matched noncarriers were examined on 2 separate days as previously described [33, 34], and biochemical profiling was performed the first examination day. The study protocol was approved by the Ethical Committee of Central Denmark Region (protocol No. 1-10-72-113-12) and undertaken in accordance with the principles of the Declaration of Helsinki II. The study participants gave their written informed consent before study participation.

### Study III—A Recall-by-Genotype Study: Lipid Meal Challenge in 24 Danish Men

We recruited 24 middle-aged healthy Danish men by genotype from the population-based Health2006 cohort [35] (n = 18) and among staff at the University of Copenhagen (n = 6). Of the 24 participants, 7 were heterozygous carriers of the nepmucin Cys82 variant and 17 were noncarriers (Table 1). The carriers and noncarriers were in silico matched by age, body mass index (BMI), fasting glucose, and glycated hemoglobin A_1c_ concentrations. The genotypes of the study participants were not blinded to the examining personnel. The study was approved by the regional ethical committee of Copenhagen (protocol No. H-1-12012-136) and conducted in accordance with the principles of the Helsinki Declaration II. Written informed consent was obtained from all study participants.

**Table 1.** Baseline characteristics of studies II and III

	Study II		Study III	
	Arg82Arg	Arg82Cys	Arg82Arg	Arg82Cys
No.	20	20	17	7
Age, y	55.1 ± 9.0	55.0 ± 9.1	55 ± 12.1	52.4 ± 14.6
Weight, kg	79.7 ± 7.2	79.1 ± 9.4	93.1 ± 12.6	85.3 ± 8.7
Body fat, %	21.4 ± 3.8	21.5 ± 3.5	26.1 ± 5.4	23.3 ± 6.6
Basal metabolic rate, kcal/d	1617 ± 133	1675 ± 201	2083 ± 263.6	2000 ± 175.3
BMI	24.6 ± 1.7	24.5 ± 2.4	28.5 ± 3.9	27.5 ± 3.6
Waist-to-hip ratio	1.00 ± 0.04	0.99 ± 0.03	0.96 ± 0.06	0.99 ± 0.05
HOMA-IR^*a*^	1.27 ± 0.53	1.55 ± 0.99	2.54 ± 1.58	2.33 ± 1.12
Smoker, %	5	25	59	25
Antihypertensive treatment, %	5	10	24	25
Lipid-lowering therapy, %	0	0	0	0
Alcohol intake > 6 units/wk, %	50	55	65	37
Moderate physical activity > 4 h/wk, %	10	15	71	63

Table comprises mean ± SD for continuous variables and percentages for categorical variables (smoking, treatment status, and lifestyle).

Abbreviations: β, T-allele effect size; BMI, body mass index; HOMA-IR, Homeostasis Model Assessment of Insulin Resistance.

^*a*^HOMA-IR is calculated from levels of fasting plasma glucose (mmol/L) and fasting serum insulin (µIU/mL), shown as log-transformed effects and SE.

The participants underwent a 6-hour standardized lipid-rich meal challenge after a minimum of 8 hours overnight fasting. The meal consisted of a lipid-rich soup of water, chicken, leek, butter, and cream (80 g of saturated fat) in a volume of 675 mL. Total energy content was 4459 kJ (1065 kcal). The fat energy percentage was 66%, carbohydrate 16%, and protein 18%. A total of 1 g of acetaminophen was added in the meal to estimate gastric emptying time [36]. All participants consumed the entire meal within a 15-minute period. The participants were instructed to avoid excessive alcohol intake, smoking, and physical activity 48 hours before the examination. Height, weight, waist and hip circumferences were measured in light indoor clothes. Waist circumference was measured halfway between the rib cage and the superior iliac spine. Hip circumference was measured at the level of the major femoral trochanter. Blood samples were taken every 30 minutes during the first 2 hours and subsequently every hour during the last 4 hours of the meal challenge.

### Study IV—Interaction Between the Arg82Cys Polymorphism and Glycemic Status on Fasting Plasma High-density Lipoprotein Subclasses

The interaction between the Arg82Cys polymorphism and glycemic status on fasting plasma lipid levels was tested in 1330 Danish men and women from the ADDITION-PRO study [37], of whom 831 were normoglycemic (2-hour plasma glucose < 7.8 mmol/L, fasting blood glucose [FBG] < 6.1 mmol/L) and 499 hyperglycemic (2-hour plasma glucose > 7.8 mmol/L, FBG > 6.1 mmol/L). Fasting plasma lipids were measured using a high-throughput serum NMR platform [27]. The study protocol was approved by the ethical committee of the Central Denmark Region (No. 20000183) and undertaken in accordance with the principles of the Declaration of Helsinki II. All participants gave written informed consent [38].

### Biochemical Measurements in Studies II and III

Plasma glucose in studies II and III was measured by a glucose-oxidase method (Vitros 5600, Ortho Clinical Diagnostics). Serum insulin and C-peptide were measured by electrochemistry luminescence-immunoassay (Roche Diagnostics GmbH). Insulin resistance was estimated by the Homeostasis Model Assessment of Insulin Resistance (HOMA-IR) [39]. ApoB48 and apoB100 were measured in EDTA-plasma samples using the enzyme-linked immunosorbent assay method (ELISA; Shibayagi Co Ltd and Immuno-Biological Laboratories Co Ltd, respectively). Double standards were measured for each run. Intra-assay and inter-assay variation ranged from 3.5% to 5.6% and 2.8% to 8.6%, respectively. Plasma levels of TGs, HDLc, and total cholesterol (TC) were measured on a Vitros 5.1 Chemistry System (Ortho Clinical Diagnostics). In the fasting state, LDLc levels were calculated according to the Friedewald formula and VLDLc was derived thereof [40]. Ultracentrifugation was performed by adjusting plasma (950 µL) to the density of 1.063 g/L (separation of VLDL/LDL and total HDL) or 1.12 g/L (separation of VLDL/LDL/HDL2 and HDL3) using NaBr. Ultracentrifugation was performed at 50.000 rpm at 4 °C for 16 hours using a Beckman Ti 50.3 rotor and a Beckman Optima LE-80K ultracentrifuge (Beckman Coulter Inc). Cholesterol and TGs were measured using enzymatic kits (CHOD-PAP from Roche Applied Sciences and GPO-Trinder from Sigma, respectively) in all fractions of plasma samples. The amounts of HDL2c and TGs were calculated as total HDL (d > 1.063) minus HDL3 (d < 1.12). The values were corrected for recovery of either cholesterol or TGs at each density. Postprandial plasma VLDLc and LDLc were not included as outcome traits because of their high levels of TGs.

### Genotyping

The rs72836561 (Arg82Cys) polymorphism of *CD300LG* was genotyped on the Illumina HumanExome Beadchip 12v1_A and the genotypes were called using GenCall, Genotyping module (version 1.9.4), or GenomeStudio software (version 2011.1, Illumina). Samples were excluded if they showed a low call rate, abnormal mean heterozygosity, high singleton count, non-European ancestry, sex discrepancy, or duplicate discordance. Genetic variants were excluded if they showed a low call rate, deviation from Hardy-Weinberg equilibrium, duplication, chromosome or allele mismatch, GenTrain score less than 0.6, cluster separation score less than 0.4, or a deviation in manual cluster checks. Missing genotypes were subsequently re-called using zCall and a second round of quality control was performed to exclude poor quality samples and variants.

### Statistical Analysis

Analyses were performed using R (versions 2.13.2 and 3.4.3) and GraphPad Prism (version 8.3.0). In study I, the association between Arg82Cys and blood lipids was examined by linear regression using an additive genetic model, adjusting for age, sex, and BMI. Meta-analyses of aggregate data of the association of the Arg82Cys variant with fasting HDL subclasses (studies II and III) were performed using inverse variance–weighted meta-analysis from linear regression analyses adjusted for age and BMI, using the R package *meta* and the function *forest.meta*, where heterogeneity was assessed by *I*^2^ and the *P* value for Cochran *Q* test [41]. If *P* was greater than .10 in the Cochran *Q* test, we applied a fixed-effects model. If *P* was less than .10, a random-effects model was employed. In studies II and III, a *P* value equal to or below .05 was defined as statistically significant. In study III, the associations between Arg82Cys polymorphism and postprandial plasma lipid levels were tested by linear regression analyses of the incremental area under the curve (AUC) and by unpaired *t* tests between genotype groups for specific time intervals. Incremental AUC was calculated using the trapezoidal method [42, 43]. In study IV, the interaction between the Arg82Cys variant and glycemic status (hyperglycemic vs normoglycemic) on lipid levels was tested by incorporating an interaction term in linear regression models. The results in studies I and IV were corrected for multiple testing by Bonferroni correction (*P*_CORRECTED_ = .05/17 = .003).

## Results

### Association of Arg82Cys Variant With High-density Lipoprotein Subclasses in 4522 British Men and Women

**Figure 2.**
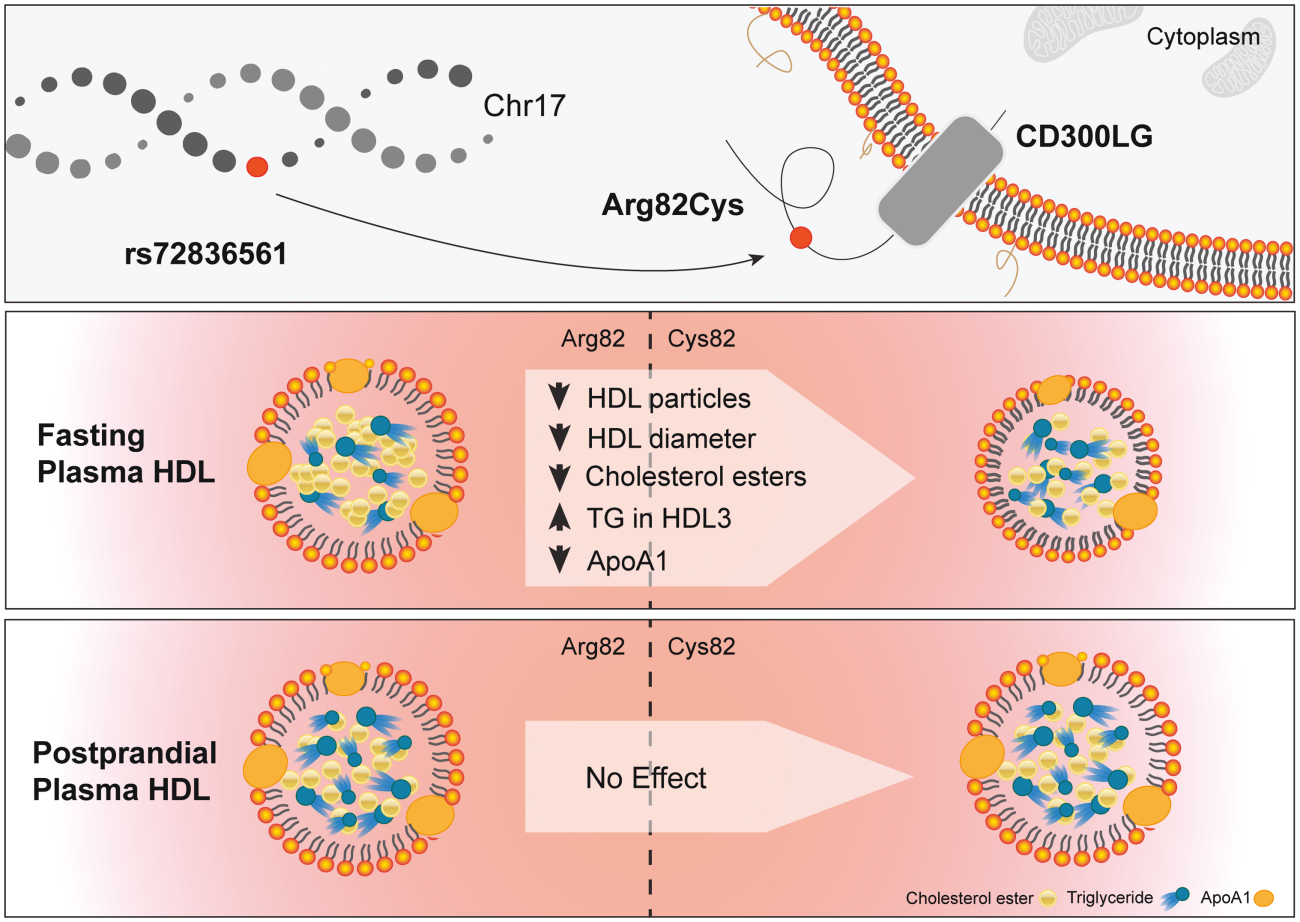
Graphical abstract of the main findings of the study. Schematic representation of the associations of the Arg82Cys polymorphism with fasting or postprandial high-density lipoprotein (HDL) subclasses and HDL composition.

To elucidate the association of Arg82Cys with lipids in HDL subclasses in a fasting state, we examined associations with HDL, HDLc, HDL cholesterol esters, and ApoA1 concentrations measured by NMR spectroscopy in 4522 men and women from the OBB cohort (study I). The results are given as effect-per-allele from the additive genetic model. We found that the Cys82 variant was associated with 0.037 mg/L lower ApoA1 levels (*P* = .008), 0.04 nm lower HDL mean diameter (*P* = .003), 0.04 µmol/L lower HDL2 cholesterol (*P* = .004), and 0.04 µM lower HDL3 cholesterol (*P* = .02) per allele (Table 2). In analyses stratified by HDL particle size, we found that the Cys82 variant was associated with 0.03 nmol/L lower concentration of M-HDL particles (*P* = .01), 0.04 nmol/L lower concentration of L-HDL particles (*P* = .003), and 0.04 nmol/L lower concentration of XL-HDL particles (*P* = .005) per allele. Similarly, the Cys82 variant was associated with 0.03 µmol/L lower concentration of M-HDLc (*P* = .02), 0.04 µmol/L lower concentration of L-HDLc (*P* = .001), and 0.03 µmol/L lower concentration of XL-HDLc (*P* = .05), as well as with 0.03 µmol/L lower concentration of M-HDL cholesterol esters (*P* = .02) and 0.05 µmol/L lower concentration of L-HDL cholesterol esters (*P* = .0004) per allele. We found no association between Cys82 carriers and concentration of S-HDL particles, suggesting that the Cys82 variant specifically reduces the levels of larger HDL particles (Fig. 2). We did not find an association with HOMA-IR, ApoB, nor LDL cholesterol (Supplementary Table S1 [44]).

**Table 2.** Association of nepmucin Arg82Cys with high-density lipoprotein subclass in plasma in study I

	Arg82Arg, mean ± SD	Arg82Cys, mean ± SD	Cys82Cys, mean ± SD	Effect per allele, β (95% CI)	P_*add*_
**Total HDL (HDL2 + HDL3)**					
ApoA1, mg/L	1.44 ± 0.25	1.40 ± 0.25	1.42 ± 0.14	–0.04 (–0.06 to –0.01)	.008
HDL cholesterol, mmol/L	1.42 ± 0.33	1.36 ± 0.34	1.33 ± 0.10	–0.04 (–0.07 to –0.02)	.001
HDL mean diameter, nm	9.83 ± 0.27	9.79 ± 0.28	9.66 ± 0.12	–0.04 (–0.06 to –0.01)	.003
**HDL2 (very large + large HDL)**					
Cholesterol, µmol/L	920.2 ± 306.8	872 ± 316.2	808.9 ± 98.05	–0.04 (–0.07 to –0.02)	.004
**Very large HDL**					
Cholesterol, µmol/L	170.7 ± 93.15	157 ± 94.05	165.4 ± 65.05	–0.03 (–0.06 to 0.0007)	.05
Cholesterol esters, µmol/L	125.1 ± 65.23	115.5 ± 65.16	129.8 ± 39.04	–0.02 (–0.05 to 0.0035)	.1
Particle concentration, nmol/L	289.2 ± 175.2	265.9 ± 184.3	204.3 ± 109	–0.04 (–0.06 to –0.01)	.005
**Large HDL**					
Cholesterol, µmol/L	315.3 ± 188.3	291.9 ± 195.6	216.4 ± 112.8	–0.04 (–0.07 to –0.02)	.001
Cholesterol esters, µmol/L	248.6 ± 144.6	227.9 ± 151.7	178.3 ± 96.68	–0.05 (–0.08 to –0.03)	.0004
Particle concentration, nmol/L	887.2 ± 441.6	821.3 ± 450.3	598.8 ± 176.2	–0.04 (–0.07 to –0.02)	.003
**HDL3 (medium + small HDL)**					
Cholesterol, µmol/L	523.8 ± 32.29	495.4 ± 48.96	505.1 ± 48.22	–0.04 (–0.07 to –0.01)	.02
**Medium HDL**					
Cholesterol, µmol/L	451.2 ± 105.4	434.3 ± 100.5	421.2 ± 18.82	–0.03 (–0.06 to 0.006)	.02
Cholesterol esters, µmol/L	366.1 ± 83.24	353.4 ± 80.36	343.2 ± 10.47	–0.03 (–0.06 to –0.005)	.02
Particle concentration, nmol/L	1.71 ± 0.36	1.65 ± 0.33	1.60 ± 0.10	–0.04 (–0.06 to –0.006)	.01
**Small HDL**					
Total lipids, µmol/L	1.17 ± 0.13	1.16 ± 0.12	1.25 ± 0.066	–0.009 (–0.03 to 0.02)	.7
Cholesterol esters, µmol/L	NA	NA	NA	NA	NA
Particle concentration, nmol/L	4.37 ± 0.41	4.34 ± 0.40	4.60 ± 0.21	–0.009 (–0.04 to 0.02)	1

Association of nepmucin Arg82Cys with HDL subclass in study I (Oxford BioBank). Data presented are mean ± SD. Analyses were adjusted for age, sex, and body mass index. No information on HDL3 cholesteryl esters was available.

Abbreviations: BMI, body mass index; HDL, high-density lipoprotein; NA, not available.

### Association of Nepmucin Arg82Cys With Fasting Plasma Lipid Levels and High-density Lipoprotein Subclasses in a Meta-analysis of 64 Danish Men

Based on results from study II and baseline results from study III (including 27 heterozygous Cys82 carriers and 37 noncarriers) (Tables 3 and 4), we performed a meta-analysis to test whether there are differences between Cys82 carriers and noncarriers in relation to fasting TGs, TC, LDLc, HDLc, or HDL subclasses measured with ultracentrifugation. We found that HDL2c was 0.3 mmol/L lower (*P* = .005) and the HDL2c/HDL3c ratio was 0.5 units lower (*P* = .0004) in Cys82 carriers than in noncarriers (Table 4). Furthermore, the plasma HDL3 TG was 0.01 mmol/L higher in Cys82 carriers than in noncarriers (*P* = .0003), whereas there was no difference in plasma HDL2 TG content (*P* = .1). We also found that the HDL2/HDL3 TG ratio was 1.25 units lower in Cys82 carriers than in noncarriers (*P* = .002).

**Table 3.** Association of nepmucin Arg82Cys polymorphism with fasting plasma lipid levels in studies II and III

	Study II (n = 40), β (SE)	Study III (n = 24), β (SE)	Effect, β (95% CI), *P*
**HDL cholesterol**	–0.08 (0.12)	–0.22 (0.17)	–0.12 (–0.32 to 0.07), .2
**Triglycerides** ^*a*^	0.10 (0.15)	0.11 (0.18)	0.11 (–0.12 to 0.33), .3
**Total cholesterol**	–0.29 (0.21)	0.18 (0.57)	–0.23 (–0.61 to 0.15), .2
**LDL cholesterol**	–0.27 (0.20)	0.31 (0.48)	–0.18 (–0.55 to 0.18), .3

Meta-analysis of the association of nepmucin Arg82Cys with fasting plasma lipid levels in studies II and III. A total of 37 noncarriers (study II: 20, study III: 17) and 27 carriers (study II: 20, study III: 7) were analyzed. No statistically significant heterogeneity between studies II and III was found.

Abbreviations: HDL, high-density lipoprotein; LDL, low-density lipoprotein.

^*a*^Log-transformed effects and SE.

**Table 4.** Association of nepmucin Arg82Cys with fasting plasma of triglycerides and cholesterol in high-density lipoprotein particles in studies II and III

	Study II (n = 40), β (SE)	Study III (n = 24), β (SE)	Fixed effect, β (95% CI), *P*
**Cholesterol content in HDL2**, nmol/L	–0.24 (0.11)	–0.31 (0.18)	–0.26 (–0.45 to –0.08), .005
**Triglyceride content in HDL2**, nmol/L	0.002 (0.009)	–0.005 (0.003)	–0.001 (–0.011 to 0.002), .1
**Cholesterol content in HDL3**, nmol/L	0.05 (0.04)	0.06 (0.05)	0.05 (–0.005 to 0.112), .07
**Triglyceride content in HDL3**, nmol/L	0.007 (0.005)	0.008 (0.002)	0.01 (0.004 to 0.012), .0003
**Cholesterol ratio**, HDL2/HDL3	–0.74 (0.26)	–0.45 (0.20)	–0.57 (–0.88 to –0.25), .0004
**Triglyceride ratio**, HDL2/HDL3	–0.87 (0.56)	–1.64 (0.57)	–1.25 (–2.03 to –0.47), .002

Meta-analysis of the association of nepmucin Arg82Cys polymorphism with fasting content of triglyceride and cholesterol in HDL particles in studies II and III. Data from 37 noncarriers (study II: 20, study III: 17) and 27 carriers (study II: 20, study III: 7) were analyzed separately and combined. Data are based on ultracentrifugation estimated fasting levels of cholesterol and triglycerides in HDL2 and HDL3 subclasses. No statistically significant heterogeneity between study groups II and III was identified.

Abbreviations: β, mean effect size; HDL, high-density lipoprotein; LDL, low-density lipoprotein.

### Association of Nepmucin Arg82Cys Polymorphism With Postprandial Lipemia

**Figure 3.**
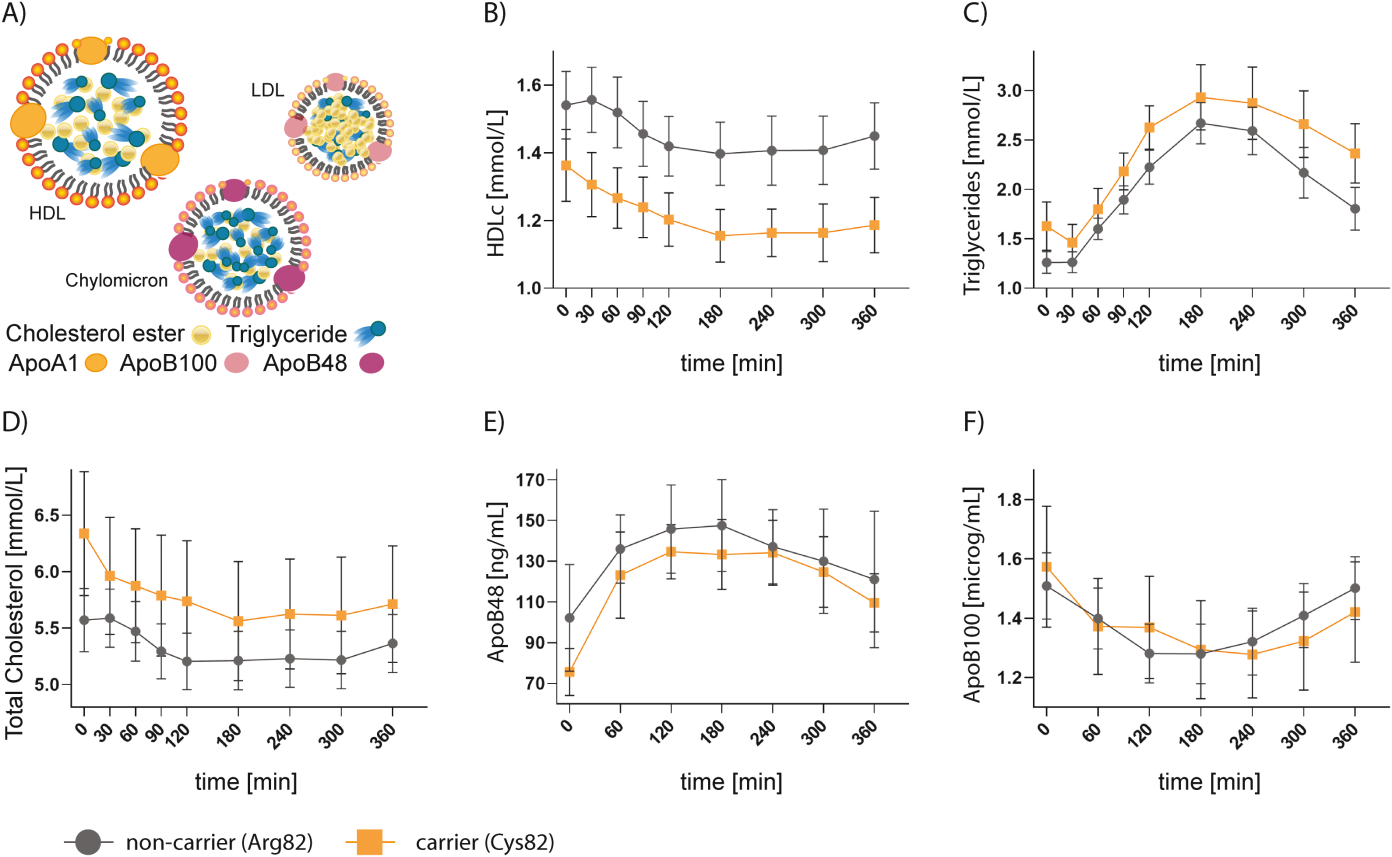
Changes in plasma levels of high-density lipoprotein cholesterol (HDLc), triglycerides (TGs), total (Tot.) cholesterol, ApoB48, and ApoB100 in nepmucin Cys82 variant carriers and noncarriers during a lipid-rich meal. The figure shows the dynamic changes of the mean ± SEM of respective traits after a meal challenge. A, Measured values (HDLc, TGs, Tot. cholesterol, ApoB48 [chylomicrons], and ApoB100 [low-density lipoprotein; LDL]). B to D, Measurements of HDLc, TGs, and Tot. cholesterol at 0, 30, 60, 90, 120, 180, 240, 300 and 360 minutes after a meal challenge. E and F, Apolipoproteins carried by chylomicrons (ApoB48) and LDL (ApoB100) at 0, 60, 120, 180, 240, 300, and 360 minutes. Gray represents noncarriers; orange represents variant carriers. The analyses were conducted in 24 (17 [Arg82Arg], 7 [Arg82Cys]) individuals from study III.

To test whether the Arg82Cys genotype affects postprandial fluctuation of blood lipids in a time-dependent manner, we examined differences between 7 heterozygous Cys82 variant carriers and 17 noncarriers in relation to AUCs for HDLc, TGs, TC, apoB48, and apoB100 (Fig. 3A) during a lipid-rich meal challenge (study III). The baseline characteristics were similar between the carriers and noncarriers of the Cys82 variant (Table 1). We found no statistically significant differences in the AUCs or lipid levels at specific time points between Cys82 carriers and noncarriers during the meal challenge (Fig. 3B-3F and Table 5).

**Table 5.** Association of nepmucin Arg82Cys polymorphism with postprandial changes in plasma lipid levels in study III

Trait	Arg82Arg, mean ± SD	Arg82Cys, mean ± SD	Effect, β (95% CI)	*P*
**HDL cholesterol iAUC** (∆mmol × L^–1^ × 360 min^–1^)	–0.7 ± 0.5	–1.2 ± 1.5	–0.5 (–1.3 to 0.3)	.2
**Triglycerides iAUC** (∆mmol × L^–1^ × 360 min^–1^)	6.0 ± 2.8	6.1 ± 3.0	0.08 (–2.5 to 2.6)	≥ .999
**Total cholesterol iAUC** (∆mmol × L^–1^ × 360 min^–1^)	–1.9 ± 1.5	–4.7 ± 5.6	–2.8 (–5.6 to 0.04)	.07
**ApoB48 iAUC** (∆ng × mL^–1^ × 360 min^–1^)	194.7 ± 185.0	283.4 ± 82.2	88.7 (–55.2 to 232.6)	.2
**ApoB100 iAUC** (∆µg × mL^–1^ × 360 min^–1^)	–0.9 ± 0.5	–1.3 ± 1.1	–0.43 (–1.1 to 0.19)	.2

Presented data are unadjusted. Analyses were conducted one in 24 (17 [Arg82Arg], 7 [Arg82Cys]) individuals from study III.

Abbreviations: HDL, high-density lipoprotein; iAUC, incremental area under the curve.

### Interaction of Arg82Cys Variant With Glycemic Status With Serum Lipid Levels

Previous studies have suggested an association of the Cys82 variant with abnormal glucose metabolism [34, 45, 46], which could be mechanistically linked to the association between Arg82Cys and lipid levels. To test whether glycemic status modifies the association between Arg82Cys and HDL subclasses, we tested the interaction with glycemic status among 1330 participants in the Danish ADDITION-PRO cohort (study IV). We stratified the participants based on 2-hour plasma glucose and FBG concentration into a normoglycemic (normal GT) group (n = 831) and a hyperglycemic (abnormal GT) group (n = 499). No statistically significant interaction between the Cys82 variant and glycemic status was found (*P* > .05 for interaction; data not shown).

## Discussion

Nepmucin is a type-I membrane protein expressed in vascular endothelial cells that shows high expression in placental tissue, skeletal muscle, and adipose tissue [23, 33, 47]. The Arg82Cys nepmucin polymorphism was originally identified in an exome-sequencing study for association with lower fasting plasma HDL cholesterol and higher fasting plasma TG concentration [14], and the association has been replicated in subsequent fasting [17, 18, 48, 49], and (for HDL) nonfasting studies [50] (Supplementary Table S2 [44]). Here, we studied association between the Cys82 polymorphism and fasting plasma concentrations of HDL and its subclasses to draw a more detailed image of its role in lipid metabolism. Furthermore, we assessed postprandial changes after a high-fat stimulus, to detect additional subtle abnormalities in lipid metabolism that could not be detected in a fasting state, and evaluated potential synergistic effects of Cys82 with glucose metabolism on fasting lipid levels.

Our analyses in the OBB cohort, applying an additive genetic model in 4522 British individuals to HDL subclasses measured by NMR spectroscopy, showed that Cys82 is associated with a smaller HDL diameter and lower cholesterol concentration in M, L, and XL-HDL particles. The associations were strongest in the L-HDL subclass. In a meta-analysis of fasting lipids from 2 RbG studies, including 27 carriers of the Cys82 polymorphism and 37 noncarriers, we observed lower cholesterol levels in plasma HDL2 particles and higher TG levels in plasma HDL3 particles of Cys82 carriers. Our results are concordant with a published GWAS of HDL subclasses measured by NMR spectroscopy in up to 24 925 individuals [17] that showed the Cys82 variant was associated with lower concentrations of L and M-HDL particles [17] (see Supplementary Table S2 [44]). Interestingly, smaller HDL diameter has been associated with adverse cardiometabolic outcomes [12, 51, 52]. In line with this, the Cys82 polymorphism reached nominal significance in the latest GWAS for cardiovascular outcomes (β = 0.057; *P* = 2.2E-3) [53] (see Supplementary Table S2 [44]) and is linked to increased atherosclerosis (*P* = 4.18E-6), and peripheral artery disease in FINNGEN (*P* = 2.73E-5/unpublished data, URL: https://r5.finngen.fi/variant/17-43848758-C-T). Furthermore, we found the Arg82 polymorphism to be linked to decreased ApoA1 level—a protein component of HDL involved in lipid metabolism (see Table 2), which has previously been linked to cardioprotective properties [11].

In our study of 24 Danish men who were challenged with an oral lipid load, we did not see a difference in postprandial lipemia between Cys82 carriers and noncarriers. While the study sample size was limited, the RbG design enhanced statistical power by including a relatively large proportion of carriers with the rare allele. We estimated to have 90% statistical power to exclude an allele-dependent reduction in postprandial HDL plasma levels exceeding 0.17 mmol/L in a test of unpaired means (α = 0.05). This may suggest that the link between Arg82Cys and lipemia is subjected to a fasting state and effects may be obscured in a postprandial state.

A previous RbG study among 42 healthy male carriers and 20 noncarriers of the Cys82 variant showed an association with lower *CD300LG* messenger RNA expression in muscle and white adipose tissue, as well as with higher intramyocellular lipid content and forearm glucose uptake [34]. Overall, the findings suggested a role for nepmucin in the regulation of glucose and lipid homeostasis. We therefore hypothesized that the association of Arg82Cys with HDL subclasses might be modified by glycemic status. However, we found no epidemiological evidence of such an interaction. These findings are supported by a previous study from 2013, in which Arg82Cys was not associated with glycemic traits (fasting glucose, 2-hour oral glucose tolerance test, glycated hemoglobin A_1c_) in a linear model [14].

Taken together, the results suggest that nepmucin is involved in lipid transport and lipoprotein maturation. As nepmucin has been shown to adhere to several polar lipids that are known to be present in HDL particles [9, 10], we speculate that nepmucin could affect the maturation of HDL molecules from the small (lipid-poor) HDL to the very large spherical HDL particle (eg, via lecithin cholesterol acyltransferase, cholesterol ester transfer protein, and hepatic lipase) [7, 54]. Also, the low levels of ApoA1 in carriers of Arg82Cys may indicate decreased uptake of cholesterol and other lipids in HDL (and thus the general decrease in HDL content) or it may simply reflect smaller HDL particles [55]. Because ApoA1 is a cofactor for lecithin cholesterol acyltransferase and thus is involved in the formation and reverse transport of cholesterol esters, specific binding assays between nepmucin and HDL are needed to confirm the mechanistic basis for the link between nepmucin and HDL metabolism. Considering previous studies that suggested a reduction of HDL2 and a shift toward smaller HDL subclasses is linked to obesity [56], it may also be of interest to focus on an obese population in future studies.

### Conclusion

Taken together, our results indicate an association between the Arg82Cys variant and a lower concentration of HDL particles and HDLc, especially in larger HDL subclasses, and lower ApoA1 level, suggesting a link between nepmucin and HDLc metabolism.

## Data Availability

Some data generated or analyzed during this study are included in this published article or in the data repositories listed in “References” [44]. Supplementary Table S1 contains associations of Arg82Cys with HOMA-IR, APOB, and LDL in study I. Supplementary Table S2 contains associations of Arg82Cys from previously published GWAS. Otherwise, restrictions apply to the availability of some data generated or analyzed during this study to preserve patient confidentiality or because they were used under license. The corresponding author will on request detail the restrictions and any conditions under which access to some of the data may be provided.

## Abbreviations

AUC: area under the curve
BMI: body mass index
FBG: fasting blood glucose
GWAS: genome-wide association studies
HDLc: high-density lipoprotein cholesterol
HOMA-IR: Homeostasis Model Assessment of Insulin Resistance
Ig: immunoglobulin
L: large
LDLc: low-density lipoprotein cholesterol
M: medium
NMR: nuclear magnetic resonance
OBB: Oxford BioBank
RbG: recall-by-genotype
S: small
TC: total cholesterol
TGs: triglycerides
VLDL: very low-density lipoprotein
XL: very large
